# Cultural and Contextual Drivers of Triple Burden of Malnutrition among Children in India

**DOI:** 10.3390/nu15153478

**Published:** 2023-08-06

**Authors:** Shri Kant Singh, Alka Chauhan, Santosh Kumar Sharma, Parul Puri, Sarang Pedgaonkar, Laxmi Kant Dwivedi, Lindsey Smith Taillie

**Affiliations:** 1 International Institute for Population Sciences, Mumbai 400088, India; drsarangpedgaonkar@gmail.com (S.P.); laxmikdwivedi@gmail.com (L.K.D.); 2 International Food Policy Research Institute (IFPRI), Delhi 110012, India; alka.chauhan@cgiar.org; 3 The George Institute for Global Health, New Delhi 110025, India; santoshiips88@gmail.com (S.K.S.); parulpuri93@gmail.com (P.P.); 4 Carolina Population Center, Department of Nutrition, University of North Carolina, Chapel Hill, NC 27516, USA; taillie@unc.edu

**Keywords:** children, malnutrition, India, overweight, stunting, anaemia

## Abstract

This study examines malnutrition’s triple burden, including anaemia, overweight, and stunting, among children aged 6–59 months. Using data from the National Family Health Survey-5 (2019–2021), the study identifies risk factors and assesses their contribution at different levels to existing malnutrition burden. A random intercept multilevel logistic regression model and spatial analysis are employed to identify child, maternal, and household level risk factors for stunting, overweight, and anaemia. The study finds that 34% of children were stunted, 4% were overweight, and 66% were anaemic. Stunting and anaemia prevalence were higher in central and eastern regions, while overweight was more prevalent in the north-eastern and northern regions. At the macro-level, the coexistence of stunting, overweight, and anaemia circumstantiates the triple burden of childhood malnutrition with substantial spatial variation (Moran’s *I*: stunting-0.53, overweight-0.41, and anaemia-0.53). Multilevel analysis reveals that child, maternal, and household variables play a substantial role in determining malnutrition burden in India. The nutritional health is significantly influenced by a wide range of determinants, necessitating multilevel treatments targeting households to address this diverse group of coexisting factors. Given the intra-country spatial heterogeneity, the treatment also needs to be tailor-made for various disaggregated levels.

## 1. Introduction

Developing nations have come a long way in reducing child mortality. With improvements in income, education, and healthcare utilisation, the Asia-Pacific region has substantially reduced the under-five mortality rates [1,2]. Despite more children surviving, millions of young children in developing countries fail to thrive, as indicated by stagnation in reducing stunting, wasting and the fastest-growing rates of overweight and obesity [3,4]. It was found that child survival interventions were less efficient in countries grappling mainly with childhood malnutrition [4]. According to the National Family Health Survey (NFHS), 2019–2021, 36 percent of children under five are stunted; 32 percent are underweight; and 67 percent are anaemic. 

Malnutrition has always been a crucial public health challenge and entails high healthcare costs and increased morbidity and mortality [4]. The nutritional needs of children are unique and crucial. Large populations suffer from undernutrition, overweight, and obesity as a result of a lack of nutritious food. These forms of malnutrition often exist simultaneously and are interconnected [5,6]. In the Indian context, there are stagnant improvements in undernutrition, with growing rates of overweight and anaemia, especially among children. Thus, overall, India’s under-five population is facing an increased burden resulting from higher rates of underweight, overweight, and anaemia.

In the recent Global Nutrition Report, India was marked as likely to miss global nutrition targets by 2025. Approximately 45 percent of deaths among children under five years of age can be attributed to undernutrition [6]. Childhood undernutrition may result in irreversible long-term effects, including impaired physical growth and cognitive development [1,4,6,7]. Poor nutrition in the first 1000 days of a child’s life can also lead to stunted growth, impaired cognitive ability, and reduced school and work performance. This might further disable them from leading a socially and economically productive life at later ages [7,8].

While there has been some progress in Asian sub-continents concerning the reduction of the undernourished population from over one billion people in the 1990s to 793 million in 2015 [5,9,10,11], childhood overweight and obesity rates are rising. In the past 40 years, the obesity pandemic has changed malnutrition patterns globally. Furthermore, overweight exhibits an increasing trend with a notable prevalence among lower-income groups, where being underweight is already a major challenge [3]. Therefore, more than two indicators of malnutrition may coexist in various parts of the Indian subcontinent. The triple burden of malnutrition among Indian must be investigated in order to inform comprehensive action against childhood malnutrition in all its forms.

Malnutrition among children is multifaceted; the mother’s nutritional status is the most important factor causing childhood malnutrition. Increased maternal education helps to reduce cases of childhood malnutrition. Children of uneducated mothers showed a greater risk of malnutrition than their counterparts [12,13]. It has been reported that girls are more susceptible to malnutrition than boys. It was seen that boys were timely fed and continued to drink mother’s milk than girls, which in turn led to health issues [14]. Higher birth order children are more prone to malnutrition than those born in lower birth orders. Children of higher order were found to be affected by severe cases of malnutrition. It has been found that mothers with many children do not have time to feed and take care of them. Shorter birth intervals do not allow the mother to regain her health properly; this results in the deteriorating health of both mothers and their children [15]. Thus, it is essential to understand these disparities in order to ensure that policies are developed to address nutrition problems among the most vulnerable sections of society.

The existing literature has adopted different approaches to define malnutrition in India. Some studies employed mother–child dyads; others highlighted the clustering of sibling malnutrition [16,17]. Other studies have focused on the intra-household burden of malnutrition based on two or more individuals affected by under- or over-nutrition [18,19]. However, only a few have captured these contrasting dimensions of malnutrition holistically for children at population-level in India. Being a highly diverse country, it is necessary to capture contrasting forms of malnutrition at varied levels in India. In addition, existing studies fail to holistically capture the emerging burden at disaggregated levels. The present study utilises three indicators of malnutrition, stunting, overweight, and anaemia, among children aged 6–59 months in India. Furthermore, the study focuses on identifying the risk factors of stunting, overweight, and anaemia, as well as assessing the contribution at various hierarchical levels to the existing burden of malnutrition.

## 2. Materials and Methods

### 2.1. Participants

The data from the fifth round of the National Family Health Survey (NFHS-5), 2019–2021, India, are used in this study. NFHS is designed to provide national and sub-national estimates of various critical indicators to monitor the SDGs on population, health, nutrition, and gender equality, among others. NFHS-5 included data from 636,699 households with 724,115 eligible women aged 15–49 years, 101,839 men, and 230,978 children under the age of five years [20].

We used a combined dataset for the current study that contained data on 219,399 children under the age of five from kid and household member files. Anaemia was one of the three markers of malnutrition that was specifically examined in children aged 6–59 months; therefore, we restricted the analysis to 198,335 children age 6–59 months.

### 2.2. Measurements

#### 2.2.1. Outcome Variables

Three anthropometric indicators of nutritional status among children, namely, stunting (height-for-age), overweight (weight-for-age), and anaemia, were used as the dependent variables in this study. 

Height-for-age is a measure of linear growth retardation and cumulative growth deficits. Children whose height-for-age z-score is below minus two standard deviations (−2 SD) from the median of the reference population are considered short for their age (stunted) or chronically undernourished [20]. Weight-for-age is a composite index of height-for-age and weight-for-height. It takes into account both acute and chronic malnutrition. Children whose weight-for-age z-score is below minus two standard deviations (−2 SD) from the median of the reference population are classified as underweight, while children whose weight-for-height z-score is more than two standard deviations (+2 SD) above the median of the reference population are considered overweight. Anaemia is a condition marked by low levels of haemoglobin in the blood. For this study, any form of anaemia among children aged 6–59 months was marked by haemoglobin levels (haemoglobin levels are adjusted for the altitude in enumeration areas above 1000 m) below 11 g/dL [20].

#### 2.2.2. Explanatory Variables

The study explores the burden of selected indicators of malnutrition in a nested set-up. The study included child, household, and mother level characteristics. These included child’s age (in months), child’s sex, birth size, birth order, place of residence, religion, caste, wealth, mother’s age, education, body mass index (BMI), and mass media exposure. In addition, for childhood anaemia, mothers’ anaemia status was also included as a predictor. 

### 2.3. Statistical Analysis

A number of hierarchical levels can be used to quantify the extent of malnutrition in the nation. The first part of this study focuses on the spatial heterogeneity of the selected indicators among 707 districts across the nation. We first created thematic maps to assess the prevalence of malnutrition at the sub-regional level. To investigate the spatial dependence and clustering of malnutrition indicators, we estimated the univariate Local Indicator of Spatial (LISA) cluster, significance maps, and Moran’s I statistics. We identified areas with statistically significant clustering of hotspots (high levels of malnutrition indicators) and cold spots (low levels of malnutrition indicators). This gave us within-country information on high performing and low performing regions with respect to geographical proximity. The computation of the spatial autocorrelation indices required spatial weights of order one, which were produced using a Queen’s contiguity weight matrix. Weights based on contiguity from the polygon boundary file were calculated using Queen’s contiguity matrix. The study proceeded with descriptive statistics to describe the sample characteristics. Furthermore, a bivariate analysis was carried out to explore the pattern of indicators of malnutrition by selected background characteristics. These values were supplemented with chi-square *p*-values to identify the statistical significance of the associations. 

It is worth mentioning that the NFHS dataset follows a hierarchical structure, where children were nested within a household, households were nested within a PSU, and PSUs were nested within districts. Thus, to account for the nested data structure, a multi-level logistic regression analysis was employed to identify the possible risk factors and to assess the contribution of selected analytical levels on malnutrition. For the present analysis, a four-level random intercept logistic regression model was applied. The Four-level random intercept logistic model has been specified for the probability of a child aged 6–59 months *i* in the HH *j*, in PSU *k* and district *l* being stunted (Y_*ijkl*_ = 1), and the same model is repeated to get results for overweight and anaemia [21].

Logit (π_*ijkl*_) = β_*o*_ + B*X*_*ijkl*_ + (*f_*0*k_* + *v_*0*jk_* + *s_*0*jkl_* + *u_*0*ijkl_*) (1)

This model estimates the log odds of π_*ijkl*_ adjusted for vector (*X*_*ijkl*_) of explanatory variables measured at the individual level. The parameter β_*o*_ represents the log odds of the malnutrition for a child belonging to the reference category of all the categorical variables. The random effect inside the brackets is interpreted as residual differential for the district *l* (*f_*0*l_*), PSU *k* (*v_*0*kl_*), HH *j* (*s_*0*jkl_*), and individual *i* (*u_*0*ijkl_*) assumed to be independent and normally distributed with mean 0 and variance $\sigma_{f0}^{2}$, $\sigma_{v0}^{2}$, $\sigma_{s0}^{2}$ and $\sigma_{u0}^{2}$, respectively. The variances quantify between districts, between PSU, between households’ variations, respectively, in the log-odds of children aged 6–59 months having stunting, overweight or anaemia. The results were presented in the form of adjusted odds ratios (AORs).

The analysis was performed using STATA Version 17.0 (StataCorp^TM^, College Station, TX, USA). Geo-Da version 1.20.0.814 (Teknowledgist^TM^, New York, NY, USA) was used to complete spatial analysis. All the estimates provided in this study are derived by applying appropriate sampling weights computed by the Demographic and Health Survey (DHS) India, 2019–2021.

## 3. Results

### 3.1. Triple Burden of Malnutrition among under Five Children in India

**Figure 1:**
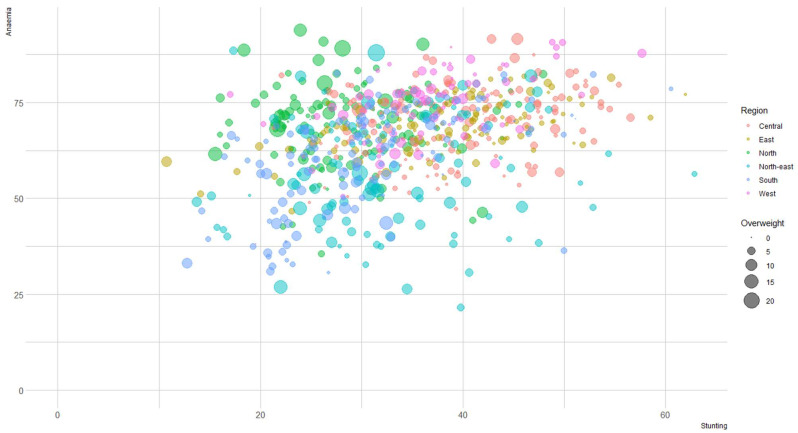
Patterns of stunting, overweight and anaemia among children under 6–59 months across 707 districts in India, National Family Health Survey (NFHS-5), 2019–2021.

Figure 1 represents the triple burden of malnutrition, stunting (*x*-axis), anaemia (*y*-axis), and overweight (*z*-axis bubble size) among children aged 6–59 months across 707 districts in India. Findings suggest that in 2021, 34 percent of the children were stunted, 4 percent were overweight, and 66 percent were anaemic. In the figure, districts are segmented into six regions, central (orange), east (light blue), north (green), north-east (yellow), south (dark blue), and west (pink). Findings illustrate that districts hailing from the central and eastern regions have a higher prevalence of stunting and anaemia but a lower burden of overweight. Children hailing from the northern region have a higher proportion of overweight, with higher prevalence of anaemia. The findings present that anaemia burden is a growing concern among children, as around 90 percent (635 out of 707) of districts had a prevalence greater than 50 percent.

**Figure 2:**
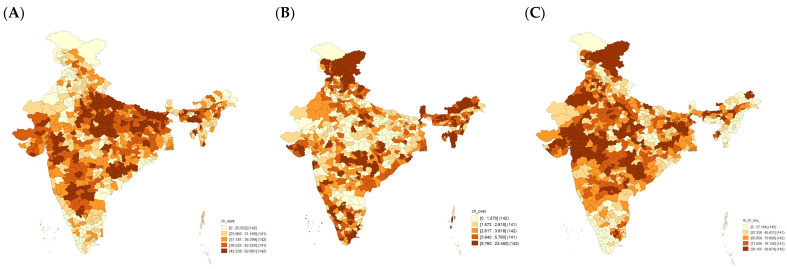
District distribution of the rates of malnutrition (**A**) stunting, (**B**) overweight, and (**C**) anaemia among children under 6–59 months across 707 districts in India, National Family Health Survey (NFHS-5), 2019–2021.

Figure 2 illustrates the distribution of stunting, overweight, and anaemia across 707 districts in India. In case of stunting, 339 districts (47.9% of the districts) had a prevalence greater than the national average (~34%). Out of 339, 105 (~31%) of the districts belonged to the central region; 81 districts (~24%) belonged to the eastern region; 49 districts (~15%) belonged to the western region; 42 districts (12%) belonged to the north-eastern region; 32 districts (~9%) belonged to the southern region; and 30 districts (~9%) belonged to the northern region in the country.

For overweight, 270 districts (38.1% of the districts) had a prevalence greater than the national average (~4%). Out of 270, 71 districts (26.3%) belonged to the north-eastern region; 61 districts (22.6%) belonged to the northern region; 51 districts (7.2%) belonged to the southern region; 34 districts (12.6%) belonged to the central region; 30 districts (11.1%) belonged to the western region; and 23 districts (8.5%) belonged to the eastern region in the country.

For anaemia, 409 districts (57.9% of the districts) had a prevalence greater than the national average (66.4%). Out of 409, 95 districts (23.2%) belonged to the central region; 91 districts (22.2%) belonged to the northern region; 74 districts (18.1%) belonged to the eastern region; 62 districts (15.2%) belonged to the western region; 52 districts (12.7%) belonged to the southern region; and 35 districts (~5%) belonged to the north-eastern region of the country.

### 3.2. Spatial Heterogeneity in the Triple Burden of Malnutrition

**Figure 3:**
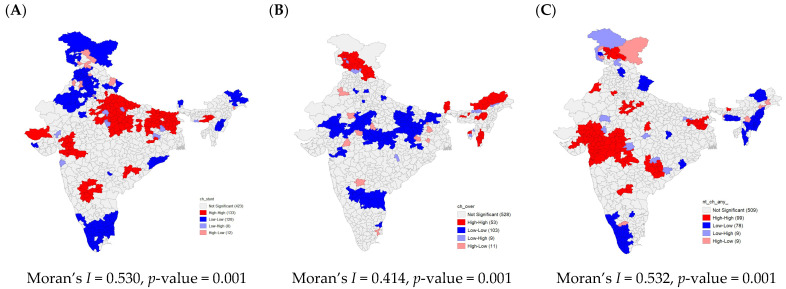
Univariate LISA clustering of (**A**) stunting, (**B**) overweight, and (**C**) anaemia among children aged 6–59 months across 707 districts in India, National Family Health Survey (NFHS-5), 2019–2021.

Figure 3A–C present findings from univariate LISA maps along with univariate Moran’s *I* statistics. In addition, significance maps for all the three indicators are presented in Figure S2. These findings depict the spatial clustering and extent of spatial autocorrelation for three indictors of malnutrition, stunting, overweight, and anaemia. All three outcomes of malnutrition show a considerably high spatial autocorrelation. There is high spatial clustering for stunting (Moran’s *I* = 0.53, *p* value = 0.001) with 133 ‘hotspots’ and 128 ‘cold spots’. Most ‘hotspots’ are located in the states of Uttar Pradesh and Bihar.

There is statistically significant clustering for overweight (Moran’s *I* = 0.41, *p* value = 0.001), with 53 ‘hotspots’ concentrated in the northern and north-eastern regions and 103 ‘cold spots’ in the central region. Spatial clustering is higher for anaemia (Moran’s *I* = 0.53, *p* value = 0.001) and identified 99 ‘hotspots’ concentrated in the Western states of Gujarat and Maharashtra. In the present context, a hot spot depicts districts which are higher in prevalence of selected outcomes of interest (stunting, overweight, and anaemia) and are surrounded by other districts with high prevalence as well, whereas a cold spot depicts districts with low prevalence surrounded by a district with lower prevalence. 

### 3.3. Description of the Study Population

Table 1 provides the descriptive statistics of the child, household, and mother level characteristics. Findings suggest that 45 percent of the study population was composed of children aged 36–59 months, 52 percent were males, and 39 percent were first in birth order. Around 73 percent of the population resided in the rural areas; 79 percent belonged to the Hindu households; 43 percent belonged to other backward classes; and 46 percent belonged to poor households.

Furthermore, Table 1 presents the burden of selected indicators of malnutrition by background characteristics. Findings suggest that for stunting, all child, household, and mother level characteristics were found to be significantly associated. Stunting was found to be higher among children aged 24–35 months (38.2%), males (37.0%), and birth order of four or more (43.8%). Stunting was higher for children residing in rural areas (38.4%), following Muslim faith (37.9%), being from scheduled castes/tribes (40.8%), and belonging to the poorest wealth quintile (47.9%). Stunting was higher among children whose mothers’ age at their birth was below 19 years (40.9%), who had no education (47.8%), who were underweight (44.4%), and who had no exposure to mass media (45.6%). 

Overweight was found to be higher among children aged 6–23 months’ (4.4%), males (3.3%), and birth order of one (3.7%). Overweight was higher for children residing in urban areas (4.1%), following Muslim faith (3.9%), being from other caste categories (4.0%), and belonging to the richest wealth quintile (4.7%). Overweight was higher among children whose mothers’ age at their birth was 25–49 years (4.2%), who had higher education (4.6%), who were overweight/obese (4.2%) and who had any exposure to mass media (3.4%).

Anaemia was found to be higher among children aged 6–23 months (4.5%) and birth order four or more (70.8%). Anaemia was higher for children residing in rural areas (69.3%), following faith other than Hindu/Muslim or Christianity (71.5%), being from scheduled castes/tribes (71.6%), and belonging to the poorest wealth quintile (72.7%). Anaemia was higher among children whose mothers’ age at their birth was below 19 years (72.3%), who had no education (72.8%), who were underweight (72.9%), who had no exposure to mass media (72.1%), and who had anaemia (73.0%). 

### 3.4. Multi-Level Regression Analysis

Table 2 presents the findings from the multi-level regression analysis showing the effects of fixed-effect and random-effect parameters. From the null model, if the variance estimate is greater than zero, it indicates that there are area differences in the selected indicators of nutrition among children aged 6–59 months. It is worth mentioning that all the selected outcomes are binary in nature; thus, a multilevel logistic regression model was considered appropriate for further analysis. The results as presented as adjusted odds ratio (AOR) for stunting, overweight, and anaemia.

Considering the final model, the intra-class correlation coefficient (ICC) indicated that 31.2 percent of the total variability in stunting is due to differences across households, followed by PSUs (10.7%) and districts (2.4%). In the random effect part, the *p*-value of the log-likelihood ratio test (LR) versus logistic regression is <0.0001 for all three indicators. This suggests that variance in selected indicators of malnutrition differs significantly across household, PSU, and district level.

Model 3 represents the findings after controlling for all child, mother, household, and community-level characteristics. All explanatory factors were found to be statistically significantly associated with stunting. Among them, children aged 36–59 months were more likely to be stunted as compared to children in the age group 6–23 months. Female children are 14 percent less likely to be stunted than their male counterparts. Children with birth order 4 or above are approximately 25 percent more likely to be stunted as compared to first birth order children. Children whose birth size was normal or above average were 31 percent less likely to be stunted as compared to their counterparts. In household level factors, children residing in rural areas were more likely than those from urban areas. Children belonging to households’ following Muslim faith were 13 percent more likely to be stunted as compared to those belonging to Hindu households. Children belonging to others caste category were 25 percent less likely to be stunted as compared to scheduled castes/tribes. Children from the richest wealth quintile households were 55 percent less likely to be stunted as compared to the poorest households. Moving on to mother-level factors, as the mother’s age increases, the chances of being stunted are reduced. Similarly, children whose mothers have attained higher education are 33 percent less likely to become stunted than children whose mothers have no education. Children of overweight mothers were 22 percent less likely to be stunted as compared to those of thin mothers. Children of mothers with any mass media exposure were 9 percent less likely to be stunted as compared to those with no exposure.

For childhood overweight, except for the mother’s age at birth and mass media exposure, all other variables were found to be statistically significant. Among them, children aged 36–59 months were 51 percent less likely to be overweight as compared to children in the age group 6–23 months. Female children are 8 percent less likely to be overweight than their male counterparts. Children with birth order four or above are approximately 17 percent less likely to be overweight as compared to first birth order children. Children whose birth size was normal or above average were 27 percent more likely to be overweight as compared to their counterparts. In household level factors, children residing in rural areas were 13 percent less likely to be overweight than those from urban areas. Children belonging to households’ following Christian faith were 29 percent more likely to be overweight as compared to those belonging to Hindu households. Children belonging to other backward class categories were 12 percent less likely to be overweight as compared to scheduled castes/tribes. Children from the richest wealth quintile households were 48 percent more likely to be overweight as compared to the poorest households. Moving on to mother-level factors, as the mother’s age increases, the chances of being overweight increases. Similarly, children whose mothers have attained secondary education are 12 percent less likely to become overweight than children whose mothers have no education. Children of overweight mothers were 92 percent more likely to be overweight as compared to thin mothers.

For anaemia, children aged 36–59 months were 70 percent more likely to be anaemic as compared to children in the age group 6–23 months. Children with birth order four or above were approximately 8 percent more likely to be anaemic as compared to first birth order children. Children whose birth size was normal or above average were 6 percent less likely to be anaemic as compared to their counterparts. In household level factors, children residing in rural areas were four percent more likely to be anaemic than those from urban areas. Children belonging to households’ following Muslim faith were 7 percent more likely to be anaemic as compared to those belonging to Hindu households. Children belonging to other backward class categories were 16 percent less likely to be anaemic as compared to scheduled castes/tribes. Children from the richest wealth quintile households were 21 percent less likely to be anaemic as compared to the poorest households. Moving on to mother-level factors, children whose mothers have attained higher education are 19 percent less likely to become anaemic than children whose mothers have no education. Children of overweight mothers were 14 percent less likely to be anaemic as compared to thin mothers. Children of mothers with any mass media exposure were 5 percent less likely to be anaemic as compared to those no exposure. Children of anaemic mothers were 66 percent more likely to be anaemic as compared to their counterparts.

## 4. Discussion

The results show that the country is dealing with a triple nutritional burden, as seen by the concurrent prevalence of stunting (34%), overweight (4%), and anaemia (66%). Further research has revealed regional variation in three indicators of malnutrition among children aged 6–59 months in 707 districts of India. The results of multi-level regression show that child, mother, and household-level variables can influence the prevalence of malnutrition in India, with the level of households explaining the most variability.

Children who were aged 36–59 months, having a birth order of four or higher, and had a birth size below normal were found to have increased rates of stunting and anaemia. Children who lived in rural areas, were Muslims, belonged to scheduled caste groups, and were from the poorest households were more likely to be stunted and anaemic. Children of young, uneducated and thin mothers were more likely to be anaemic and stunted. Children born to anaemic mothers were also more prone to be anaemic. Children aged 6–23 months who were male, having the first birth order, and whose birth size was normal or above average were more likely to be overweight. Children living in urban areas, adhering to the Christian faith, and belonging to the richest quintile were more prone to be overweight. Additionally, children of older (35–49 months), uneducated, and overweight mothers were more likely to be overweight. 

### 4.1. Possible Mechanism of the Findings

The current study has uncovered differences in malnutrition across different areas and emphasized that the central and eastern regions experience a higher prevalence of stunting and anaemia. In contrast, overweight is more common in the northern and north-eastern districts of the country. Earlier research from India has also pointed out the central and eastern regions as hotspots for stunting and anaemia [22,23]. Additional research is necessary to identify the specific contextual and policy-related factors contributing to these persistent regional disparities. Table S1 presents selected contextual determinants of malnutrition at a broader level, offering opportunities for tailored initiatives to address the nation’s subnational inequities. The variations observed in different regions can be attributed to the diverse socioeconomic, cultural, and political systems that influence dietary practices in each area.

The results of this study show that firstborn children have a height advantage over those with birth orders of four or more. It can be concluded that, negative birth-order effect stunting. These results can be supported by Dhingra and Pingali (2020), who found that children in the third (or higher) birth order have a height-to-age gap that is twice as large as that of children in the second birth order. However, these linkages hold true only in the cases where the birth spacing is less than three years. The study reaffirms that negative birth-order effects are monitored via the channels of birth spacing [24]. The instance of overweight, however, showed a positive birth order effect and suggested a greater burden for first birth order children. The firstborn child may receive superior feeding than other siblings, which could account for this. Table S2 shows the result of quantile regression with proportion of children who were stunted, underweight, and anaemic by some selected characteristics in 707 districts of India, NFHS-5 (2019–2021).

Our research shows a link between undernutrition (stunting and anaemia) and low birth weight, on the other hand, normal or above-average weight children were more likely to be overweight. This is consistent with research done in low- and middle-income nations, which showed that children born with a certain birth weight or size is likely to remain that way throughout their early life [25]. Numerous mechanisms can be underlying for undernutrition and low birth weight. First, there is a positive correlation between poverty and low birth weight, which is also known as having a connection to anthropometric growth failures and micronutrient deficiencies. Second, low birth weight is associated with an increased risk of problems and morbidities, including respiratory infections, lethargy, appetite loss, diarrhoea, jaundice, and chronic lung diseases. Greater morbidity coupled with lower birth weight results in poor physical development and micronutrient deficiencies among children [25]. Findings identified maternal factors associated with at least one indicator of nutritional status of children aged 6–59 months. These include education and mothers’ nutritional status. Further, the likelihood of stunting was lower among the children of mothers having lower age at first birth. This finding is consistent with earlier research showing a relationship between mother’s age and better child feeding [26]. Numerous more studies relate mother’s age to better child nutrition through avenues of increased autonomy and empowerment [27]. 

Historically, studies have shown that maternal education and nutritional status have a significant role in driving the nutritional status of children [21,28]. The regions marked by low female education have a higher prevalence of malnutrition among children, especially undernutrition [27]. This finding corroborates with the existing literature. Children born to educated mothers have lower risk of stunting, overweight, and anaemia, and thus, malnutrition was found to be significantly negatively associated with women’s educational attainment [28].

Poor maternal nutrition has been established as an important public health issue for both mothers and their children. Malnutrition among mothers is found to be linked with increased maternal morbidity, preterm deliveries, and small-for-gestational-age babies [2,28]. These babies are at a higher risk of anthropometric failure and micro-nutrient deficiencies [29]. According to existing research, numerous factors contribute to the link between childhood and maternal anaemia. In addition to premature birth and a child’s weight, anaemia has detrimental effects on the iron content of breast milk, which eventually causes childhood anaemia [28]. It is important to note that research in the field indicates a positive relationship between empowerment and exposure to the media. This could possibly be the reason why exposure to the media, which inculcates knowledge and increases awareness has a positive impact on reducing malnutrition [27].

While overweight was more common among wealthy household members, stunting and anaemia were more common among the poorest households. Existing research based in India has shown that households from low socioeconomic backgrounds are compelled to live with severe food insecurity; as a result, they eat items low in nutrients and calories. Additionally, they may not have enough money to effectively feed their kids, which leads to malnutrition. The children of wealthy families become overweight because they have ample resources and are more inclined to afford to feed their children unhealthy packaged foods that are high in sugar, fat, and sodium.

Distinction between urban and rural settings can be seen in terms of the type of malnutrition. Previous studies have used the greater accessibility of healthcare services in urban settings to explain it. The higher prevalence of stunting and anaemia in rural areas can be explained through the pathways of poverty, but in the context of India, this discrepancy can also be partially attributable to socioeconomic inequalities between rural and urban areas [22,30,31].

Dealing with malnutrition in India is particularly difficult compared to other nations since it is deeply ingrained in the social structure. It is clear that stunting and anaemia are more common in children from scheduled castes. Studies already conducted highlight differences in the consumption of nutrient-dense foods among socially disadvantaged groups (which includes individuals from lower castes as well). For several decades age disparities between poorer and upper castes in India have been a key cause of this inequality [32]. Additionally, dietary behaviours that are based on cultural and socioeconomic variations can be linked to differences in malnutrition due to religious differences.

### 4.2. Policy Implications of These Findings

In the past decade, malnutrition has been one of the top priorities of policymakers in India, with increasing efforts to target the affected group and improve nutritional status, especially among children. Overall, India’s strategy has been primarily focused on reducing food insecurity, improving environmental factors associated with malnutrition, and ensuring adequate nutrition. One such scheme to control malnutrition is based on schemes like the Public Distribution System (PDS). It is based on reducing food insecurity but suffers from the drawback of a disrupted distribution system, and the food distributed is primarily low in nutritional value. Such schemes might be practical to diminish food insecurity, but do not have contributed significantly for addressing the concerns related to malnutrition.

India’s primary nutritional and child development scheme, the Integrated Child Development Scheme (ICDS), has expanded steadily during its 45 years of existence. The scheme launched in 1975, now covers almost all development blocks in the country and has addressed some of the important underlying causes of undernutrition. In 2017, the government of India also launched POSHAN Abhiyaan, its flagship National Nutrition Mission, which aims to improve nutrition among children, pregnant women, and lactating mothers. It further aims to provide a convergence mechanism for the country’s response to malnutrition. Considering the environmental factors, a recent initiative, the Swachh Bharat Abhiyan, launched in 2014, is theorised to reduce stunting. However, the impact of these programs is yet to be evaluated. The present study mapped the spatial variation and identified the hot-spot and cold-spot areas based on clustering of malnutrition at district levels. These findings would be helpful to the planners and policy makers to help build new interventions for those specific underprivileged districts. In this context, the National Health Mission (NHM) initiative by the Govt. of India is working towards child and maternal health across India to improve the prevailing situation. The Govt. of India in 2017 has set up the National Nutrition Mission (NNM) for programmatic intervention with a three-year budget of INR 9046.17 crore in high priority districts of the country [33]. Despite these efforts, malnutrition among under-five children is not entirely elevated. Currently, India, on the whole, is experiencing three contrasting indicators of malnutrition; however, there is little focus on increasing rates of overweight among children. Consumption of ultra-processed foods is hypothesised to be a significant driver of overweight among children. Also, it provides little or no nutritional value and thus does not address the problem of undernutrition either. Therefore, there is a need for a more inclusive approach from the government and the individuals. 

One approach that can be adopted rests on monitoring the food environment. The existing literature establishes that the food environment is a significant driver of nutrition-related conditions; hence, it is imperative to pivot policies and interventions to encourage healthy consumption and discourage consumption of unhealthy foods. Simple interventions such as front-of-package warning labels are crucial to discourage the excess intake of sugars, fats, and sodium [31,34]. It is also important to note that consumption of excess sugar, fats, and sodium bring multi-faceted issues fuelling overweight as well as anaemia. The Nutritional Advocacy in Public Interest (NAPI) recommends the use of food fact checker to access the nutritional composition and suitability of food items before consumption.

The findings imply that the greatest variation in malnutrition is explained at the household level. Hence, policies that stimulate the food industry to create or reformulate their products’ nutritional content may impact household consumption. This indicates that intervention at this level will help achieve more significant results since children consume the majority of their foods in their households. Therefore, simple warning labels that enable easy identification of ‘good’ and ‘bad’ foods will help target the less educated population as well.

### 4.3. Strengths and Limitations

The major strength of this study is its use of a nationally representative dataset to examine the triple burden of malnutrition among children aged 6–59 months in India. The study uses disaggregated level analysis to give a comprehensive picture. However, the study has a couple of limitations. Firstly, it has not included breastfeeding and supplementary feeding practices as the markers of accessing different dimensions of malnutrition to avoid truncation of data as this information is only available for children up to 23 months. Dietary intake data or Infant and Young Child Feeding (IYCF) indicators may prove to be an important piece for future research on malnutrition among children. Secondly, the Indian DHS is a cross-sectional survey; hence, it can only support conclusions about association rather than causation, in contrast to a longitudinal study which may have a potential to look at progression from low birth weight to malnutrition within a framework of theory of change.

## 5. Conclusions

This study shows that the triple burden of malnutrition affects children aged 6–59 months in India. It also identifies the susceptible areas and subgroups of the population that primarily exhibit an elevated prevalence of one or more malnutrition indicators. The presence of several forms of malnutrition necessitates a closer examination of the current programmes to begin taking overweight into account alongside stunting and anaemia using multi-sectoral approach [35]. Continuous efforts are sought towards formulating strategies to achieve the Sustainable Development Goal of ending all forms of malnutrition by 2030.

## Figures and Tables

**Table 1:** Descriptive statistics of child, household, and mother characteristics and prevalence of stunted, overweight, and anaemia by selected background characteristics among children aged 6–59 months in India, National Family Health Survey (NFHS-5), 2019–2021.

Background Characteristics	Weighted Percentage (N = 198,335)	Stunting, %	Overweight, %	Anaemia, %
**Child Characteristics**				
**Age of children (months)**				
6–23	32.89	33.96	4.36	79.05
24–35	22.08	38.16	2.50	71.81
36–59	45.02	37.25	2.56	58.71
		Chi-square *p*-value < 0.000	Chi-square *p*-value < 0.000	Chi-square *p*-value < 0.000
**Sex of child**				
Male	51.92	37.03	3.26	68.14
Female	48.08	35.68	3.02	68.16
		Chi-square *p*-value < 0.000	Chi-square *p*-value < 0.010	Chi-square *p*-value > 0.10
**Birth Order**				
1	39.07	31.93	3.65	66.35
2 to 3	33.94	35.52	3.08	68.06
4+	27.00	43.82	2.49	70.81
		Chi-square *p*-value < 0.000	Chi-square *p*-value < 0.000	Chi-square *p*-value < 0.000
Birth Size and Weight Composite Index *				
Low	35.98	41.45	2.59	69.95
Normal or Above Average	64.02	33.39	3.47	67.11
		Chi-square *p*-value < 0.000	Chi-square *p*-value < 0.000	Chi-square *p*-value < 0.000
**Household Characteristics**				
**Place of residence**				
Urban	27.18	30.63	4.09	64.93
Rural	72.82	38.44	2.80	69.29
		Chi-square *p*-value < 0.000	Chi-square *p*-value < 0.000	Chi-square *p*-value < 0.000
**Religion**				
Hindu	79.33	36.36	3.06	68.74
Muslim	16.32	37.88	3.39	67.74
Christian	2.08	32.12	3.5	53.78
Others	2.27	30.45	3.95	71.48
		Chi-square *p*-value < 0.000	Chi-square *p*-value < 0.000	Chi-square *p*-value < 0.000
**Caste**				
Scheduled Caste/Tribes	33.00	40.83	2.78	71.59
Other Backward Classes	43.40	35.97	2.96	66.31
Others	23.60	30.80	3.99	66.61
		Chi-square *p*-value < 0.000	Chi-square *p*-value < 0.000	Chi-square *p*-value < 0.000
**Wealth**				
Poorest	24.17	47.87	2.38	72.66
Poorer	21.55	40.90	2.56	69.92
Middle	19.52	35.08	3.12	67.77
Richer	18.54	28.60	3.53	64.87
Richest	16.21	23.00	4.74	62.84
		Chi-square *p*-value < 0.000	Chi-square *p*-value < 0.000	Chi-square *p*-value < 0.000
**Mother’s Characteristics**				
**Mother’s Age at Birth (in years)**				
Below 19	34.32	40.92	2.68	72.32
20–24	49.35	35.76	3.13	66.62
25–49	16.33	28.51	4.19	63.82
		Chi-square *p*-value < 0.000	Chi-square *p*-value < 0.000	Chi-square *p*-value < 0.000
**Mother’s education**				
No education	21.23	47.75	2.57	72.83
Primary	12.28	42.88	2.61	70.5
Secondary	50.72	34.09	3.08	67.53
Higher	15.77	22.97	4.58	61.71
		Chi-square *p*-value < 0.000	Chi-square *p*-value < 0.000	Chi-square *p*-value < 0.000
**Mother’s BMI**				
Underweight	19.78	44.36	2.12	72.91
Normal	60.96	36.5	3.14	68.51
Overweight or Obese	19.26	27.71	4.17	62.02
		Chi-square *p*-value < 0.000	Chi-square *p*-value < 0.000	Chi-square *p*-value < 0.000
**Mass Media Exposure**				
No exposure	28.39	45.57	2.52	72.11
Any Media Exposure	71.61	32.72	3.39	66.57
		Chi-square *p*-value < 0.000	Chi-square *p*-value < 0.000	Chi-square *p*-value < 0.000
**Mother’s Anaemia**				
No Anaemia	40.37			60.94
Anaemia	59.63			72.99

Note. (1) Chi-square *p*-value < 0.000. (2) * Birth Size and Weight Composite Index is based on computation by the authors (see Supplementary S1). (3) BMI—Body Mass Index.

**Table 2:** Multilevel logistic regression of stunting, overweight, and anaemia among children 6–59 months in India, 2019–2021.

Background Characteristics	Adjusted Odds Ratio (95% Confidence Interval)								
	Childhood Stunting			Childhood Overweight			Childhood Anaemia		
	Model-1	Model-2	Model-3	Model-1	Model-2	Model-3	Model-1	Model-2	Model-3
**Age of children (months)**									
6–23^®^		1.00	1.00		1.00	1.00		1.00	1.00
24–35		1.27 *** (1.22, 1.31)	1.27 *** (1.22, 1.31)		0.45 *** (0.41, 0.49)	0.44 *** (0.41, 0.49)		0.60 *** (0.58, 0.62)	0.60 *** (0.58, 0.63)
36–59		1.19 *** (1.16, 1.23)	1.19 *** (1.17, 1.23)		0.49 *** (0.46, 0.53)	0.49 *** (0.46, 0.53)		0.30 *** (0.29, 0.31)	0.30 *** (0.29, 0.31)
**Sex of child**									
Male^®^		1.00	1.00		1.00	1.00		1.00	1.00
Female		0.86 *** (0.84, 0.89)	0.86 *** (0.84, 0.89)		0.92 ** (0.87, 0.98)	0.92 * (0.86, 0.98)		0.98 (0.95, 1.00)	0.98 (0.95, 1.00)
**Birth Order**									
1^®^		1.00	1		1.00	1.00		1.00	1.00
2 to 3		1.16 *** (1.13, 1.19)	1.16 *** (1.13, 1.19)		0.85 *** (0.79, 0.90)	0.84 *** (0.78, 0.89)		1.06 *** (1.03, 1.09)	1.06 *** (1.04, 1.09)
4+		1.28 *** (1.23, 1.34)	1.25 *** (1.20, 1.31)		0.83 *** (0.74, 0.93)	0.83 *** (0.74, 0.93)		1.09 *** (1.04, 1.14)	1.08 *** (1.03, 1.13)
**Birth Size/Weight**									
Low^®^		1.00	1.00		1.00	1.00		1.00	1.00
Normal or Above Average		0.68 *** (0.67, 0.71)	0.69 *** (0.68, 0.71)		1.29 *** (1.20, 1.37)	1.27 *** (1.19, 1.37)		0.94 *** (0.91, 0.96)	0.94 *** (0.92, 0.97)
**Place of residence**									
Urban^®^			1.00			1.00			1.00
Rural			1.94 ** (1.90, 1.99)			0.87 *** (0.79, 0.95)			1.04 * (1.01, 1.08)
**Religion**									
Hindu^®^			1.00			1.00			1.00
Muslim			1.13 *** (1.07, 1.18)			1.03 (0.92, 1.16)			1.07 ** (1.02, 1.13)
Christian			0.83 *** (0.77, 0.89)			1.29 *** (1.08, 1.53)			0.74 *** (0.68, 0.80)
Others			0.86 *** (0.79, 0.94)			1.56 *** (1.31, 1.86)			0.99 (0.92, 1.08)
**Caste**									
Scheduled Caste/Tribes^®^			1.00			1.00			1.00
Other Backward Classes			0.89 *** (0.86, 0.92)			0.88 *** (0.81, 0.96)			0.84 *** (0.82, 0.87)
Others			0.75 *** (0.71, 0.78)			1.04 (0.94, 1.16)			0.99 (0.92, 1.08)
**Wealth**									
Poorest^®^			1.00			1.00			1.00
Poorer			0.82 *** (0.79, 0.86)			1.07 (0.96, 1.19)			0.90 *** (0.86, 0.94)
Middle			0.71 *** (0.68, 0.74)			1.16 * (1.03, 1.30)			0.88 *** (0.84, 0.92)
Richer			0.55 *** (0.53, 0.58)			1.18 * (1.04, 1.34)			0.83 *** (0.79, 0.88)
Richest			0.45 *** (0.42, 0.48)			1.48 *** (1.27, 1.72)			0.79 *** (0.74, 0.84)
**Mother’s Age at Birth (in years)**									
15–24^®^		1.00	1.00		1.00	1.00		1.00	1.00
25–34		0.92 *** (0.89, 0.95)	0.94 *** (0.94, 1.03)		1.05 (0.97, 1.13)	1.04 (0.97, 1.12)		0.97 (0.95, 1.00)	0.98 (0.95, 1.01)
35–49		0.82 *** (0.78, 0.85)	0.86 *** (0.83, 0.89)		1.12 * (1.02, 1.23)	1.07 (0.97, 1.18)		0.97(0.93, 1.00)	0.99 (0.94, 1.03)
**Mother’s education**									
No education^®^		1.00	1.00		1.00	1.00		1.00	1.00
Primary		0.96 * (0.91, 0.99)	0.98 (0.94, 1.03)		0.97 (0.85, 1.01)	0.95 (0.84, 1.07)		0.94 ** (0.89, 0.98)	0.95 * (0.91, 0.99)
Secondary		0.71 *** (0.69, 0.75)	0.82 *** (0.79, 0.86)		0.93 (0.84, 1.03)	0.88 * (0.80, 0.98)		0.84 *** (0.81, 0.88)	0.89 *** (0.86, 0.92)
Higher		0.49 *** (0.47, 0.52)	0.67 *** (0.63, 0.71)		1.16 * (1.03, 1.32)	1.01 (0.88, 1.15)		0.73 *** (0.69, 0.77)	0.81 *** (0.77, 0.86)
**Mother’s BMI**									
Underweight^®^		1.00	1.00		1.00	1.00		1.00	1.00
Normal		0.75 *** (0.73, 0.78)	0.78 *** (0.75, 0.81)		1.50 *** (1.37, 1.65)	1.47 *** (1.34, 1.62)		0.91 *** (0.88, 0.94)	0.92 *** (0.88, 0.95)
Overweight or Obese		0.57 *** (0.54, 0.59)	0.63 *** (0.59, 0.66)		2.02 *** (1.81, 2.27)	1.92 *** (1.71, 2.15)		0.83 *** (0.79, 0.87)	0.86 *** (0.82, 0.89)
**Mass Media Exposure**									
No exposure^®^		1.00	1.00		1.00	1.00		1.00	1.00
Any Media Exposure		0.81 *** (0.78, 0.84)	0.91 *** (0.88, 0.95)		1.06 (0.98, 1.15)	1.00 (0.92, 1.09)		0.91 *** (0.88, 0.94)	0.95 ** (0.92, 0.98)
**Mother’s Anaemia**									
No Anaemia^®^								1.00	1.00
Anaemia								1.67 *** (1.63, 1.72)	1.66 *** (1.62, 1.71)
**Constant**	0.42 *** (0.401–0.435)	1.06 * (0.996–1.132)	1.31 *** (1.217–1.422)	0.01 *** (0.008, 0.012)	0.01 *** (0.006, 0.102)	0.01 *** (0.008, 0.0115)	2.25 *** (2.146, 2.361)	4.97 *** (4.600, 5.370)	5.45 *** (4.987, 5.957)
**Random intercept parameter**									
Var (district)	0.417 (0.401–0.434)	0.129 (0.112–0.149)	0.113 (0.098–0.132)	0.642 (0.549, 0.749)	0.607 (0.517, 0.713)	0.555 (0.469, 0.655)	0.37 (0.325, 0.412)	0.34 (0.301, 0.385)	0.32 (0.28, 0.36)
Var (PSU)	0.462 (0.432–0.493)	0.403 (0.375–0.434)	0.397 (0.369–0.428)	1.483 (1.337, 1.644)	1.572 (1.415, 1.747)	1.568 (1.411, 1.741)	0.48 (0.452, 0.507)	0.51 (0.482, 0.544)	0.51 (0.48, 0.54)
Var (HHs)	1.071 (0.987–1.162)	1.003 (0.920–1.094)	0.977 (0.895–1.068)	1.579 (1.277, 1.955)	1.741 (1.415, 2.142)	1.728 (1.403, 2.128)	0.14 (0.093, 0.210)	0.29 (0.231, 0.370)	0.29 (0.23, 0.37)
ICC (district) (%)	4.621	2.67	2.38	9.17	8.42	7.77	8.56	7.67	7.16
ICC (PSU) (%)	13.75	11.04	10.69	30.37	30.22	29.72	19.76	19.22	18.68
ICC (HHs) (%)	34.93	31.83	31.16	52.96	54.37	53.92	23.03	25.82	25.25
**Model fit statistics**									
Wald test X^2^		3820.63	4583.49		761.65	833.12		6778.54	6897.73
LR test vs. logistic regression: *p*-value		<0.0001	<0.0001		<0.0001	<0.0001		<0.0001	<0.0001

Note. (1) ® represents the reference category (2) *** *p* < 0.001, ** *p* < 0.05, * *p* < 0.1. (3) Model 1. Null Model; Model 2. Child + Mother Level Characteristics; Model 3. Child + Mother + Household and Community Level Characteristics. (4) Birth weight/size is a composite index based on computation by authors (see Supplementary S1). (4) PSU—Primary Sampling Unit; HH—Household; BMI—Body Mass Index.

## Data Availability

All data used for the study are publicly available on the website of the International Institute for Population Sciences Mumbai. IIPS was the nodal agency for NFHS-4 and NFHS-5; therefore, the IIPS data centre has also made use of the public’s availability of data.

## Supplementary Materials

The following supporting information can be downloaded at: https://www.mdpi.com/article/10.3390/nu15153478/s1. Figure S1. Univariate LISA significance map of (A) stunting (B) overweight and (C) anaemia among children aged 6-59 months across 707 districts in India, National Family Health Survey (NFHS-5), 2019–21. Table S1. Ordinary Least Square and Spatial Auto Regressive (SAR) models for malnutrition outcomes (a) stunting (b) overweight and (c) anaemia among children aged 6–59 months across 707 districts, India, National Family Health Survey (NFHS-5), 2019–2021. Table S2. Result of quantile regression with proportion of children who were stunted, underweight and anaemia by some selected characteristics in 707 districts of India, NFHS-5 (2019–2021). *** *p* < 0.001, ** *p* < 0.05, * *p* < 0.1.
