# Supplementary material for: Cultural and Contextual Drivers of Triple Burden of Malnutrition among Children in India
Source: Nutrients. 2023 Aug 6;15(15):3478. doi: 10.3390/nu15153478 (PMC10420920; doi:10.3390/nu15153478)
Supplement: Supplementary file 1 [file nutrients-15-03478-s001.zip › nutrients-2454246-supplementary.pdf]

Supplementary Material

Supplementary S1: Computation of composite indicator of Birth Weight and Birth Size

Birth weight is a crucial anthropometric indicator used to assess nutrition among children, and it is recognized as one of the risk factors for stunting in children under 5 years of age [27,28]. However, missing data points in the birth weight variable can pose challenges for analysis. To address this issue, we developed a composite indicator that combines both birth weight and size data.

To compute the composite indicator, we first calculated individual indicators for 'birth weight' and 'size of child at birth'. We recoded the variable "m19" to create an indicator for birth weight:

- The indicator takes the value 1 for birth weight ranging from the minimum value to 2500 grams, indicating "Low Birth Weight."
- The indicator takes the value 2 for birth weight between 2501 and 9990 grams, indicating "Normal or Above."
- Any data points labeled as 'not weighed' or 'don't know' were recoded as missing.

Similarly, we computed the birth size indicator by recoding the available variable "m18" in the NFHS dataset:

- The indicator takes the value 1 if the size of the child at birth is reported as 'smaller than average' or 'very small.'
- The indicator takes the value 2 if the size at birth is reported as 'very large,' 'larger than average,' or 'average.'
- All other values, including 'don't know,' were recoded as missing.

To create the final composite indicator, we considered all non-missing values from the birth weight variable. However, since the birth weight variable contained approximately 30,000 missing cases, we took values from the birth size indicator in those instances to ensure comparability for all cases in further analyses.

By constructing this composite indicator, we aimed to address the issue of missing data and create a robust metric that incorporates both birth weight and size, providing a more comprehensive understanding of early childhood malnutrition in our study population. This approach enables us to overcome the limitations posed by missing data and offers valuable insights for further analysis and interpretation of our findings.

A.

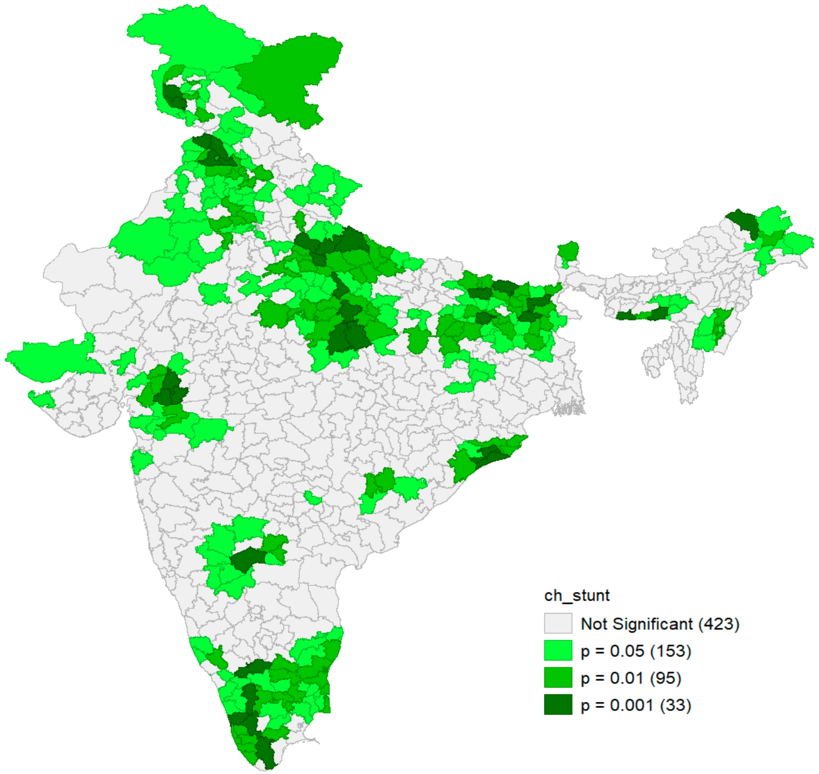

B.

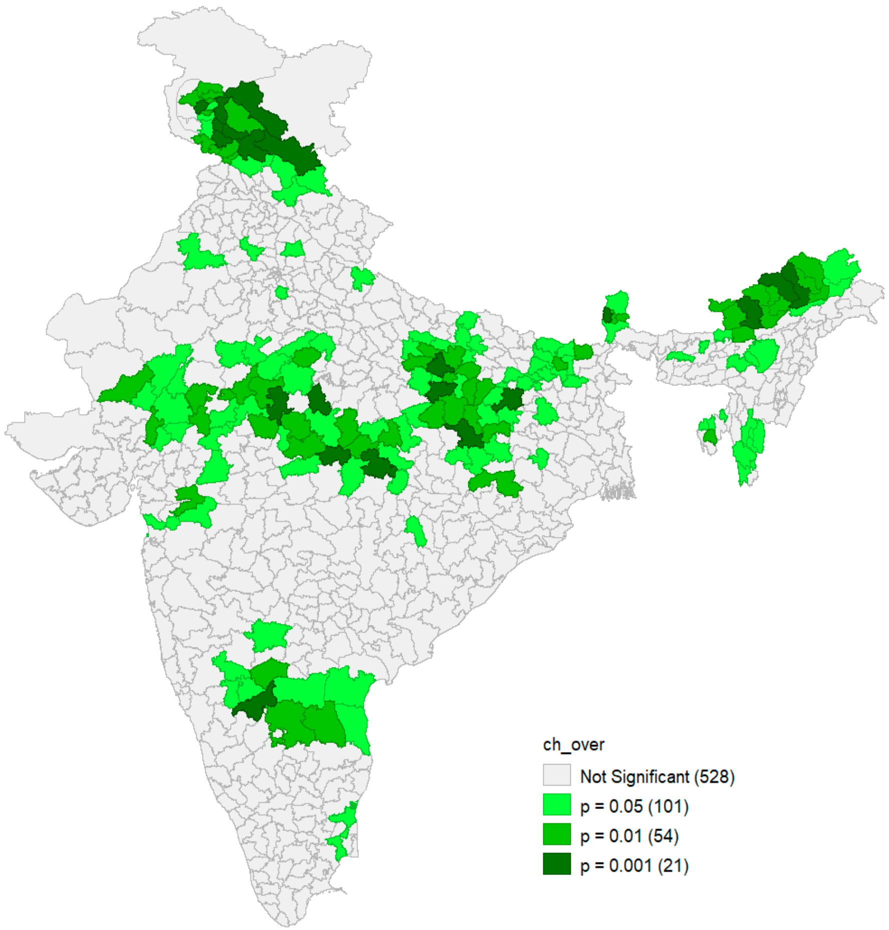

C.

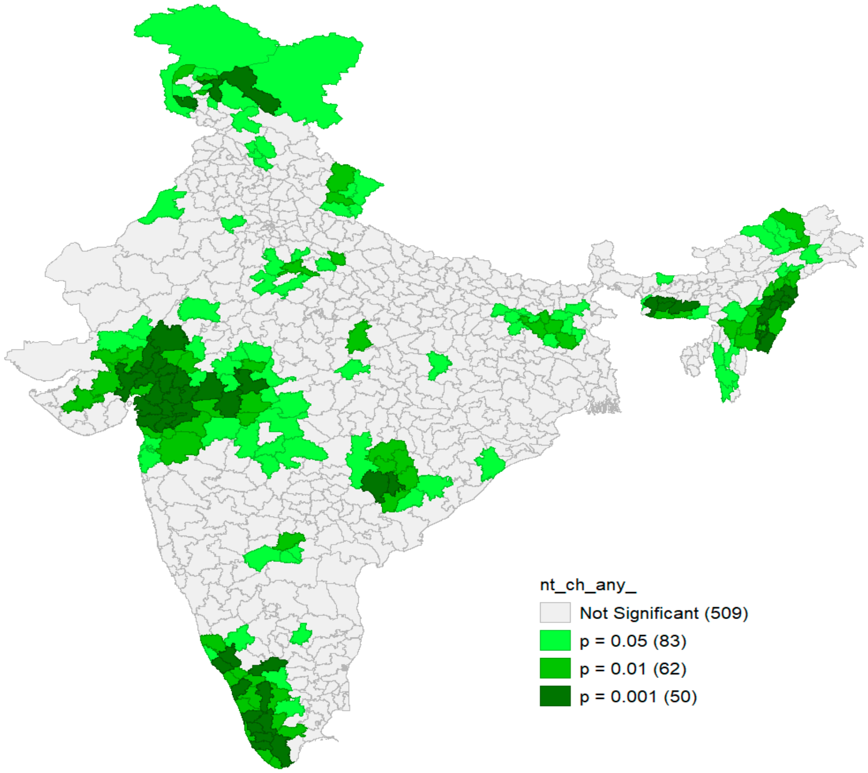

Figure S1. Univariate LISA significance map of (A) stunting (B) overweight and (C) anaemia among children aged 6-59 months across 707 districts in India, National Family Health Survey (NFHS-5), 2019-21.

Table S1. Ordinary Least Square and Spatial Auto Regressive (SAR) models for malnutrition outcomes (a) stunting (b) overweight and (c) anaemia among children aged 6-59 months across 707 districts, India, National Family Health Survey (NFHS-5), 2019–2021.

Predictors	Coefficient (p-value)		
	**Ordinary Least Square (OLS)**	**Spatial Lag Model (SLM)**	**Spatial Error Model (SEM)**
**(a) Stunting**			
Rural	-0.029 (0.029)	-0.019 (0.145)	-0.026 (0.059)
Poorest	0.046 (0.063)	0.037 (0.118)	0.066 (0.058)
Scheduled Castes/Tribes	-0.013 (0.379)	0.002 (0.914)	0.001 (0.951)
Ten or more years of Schooling	0.013 (0.527)	0.018 (0.313)	0.005 (0.827)
Exposure to Mass Media	0.004 (0.893)	0.020 (0.463)	0.024 (0.432)
Muslim	0.009 (0.521)	0.025 (0.084)	0.015 (0.390)
Female Child	0.171 (0.011)	0.142 (0.023)	0.132 (0.032)
Three or more Birth Order	0.364 (0.000)	0.290 (0.000)	0.327 (0.000)
Children under 6 months exclusively breastfed	0.009 (0.333)	0.008 (0.362)	0.009 (0.253)
Children 6-23 months receiving an adequate diet	-0.028 (0.443)	-0.010 (0.767)	-0.044 (0.238)
Women with Body Mass Index<18.5 kg/m2	0.353 (0.000)	0.277 (0.000)	0.310 (0.000)
Household with Improved Sanitation	-0.061 (0.004)	-0.052 (0.009)	-0.064 (0.008)
Constant	15.90 (0.000)	6.01 (0.122)	17.38 (0.000)
Number of Observations	707	707	707
Log likelihood	-2299.98	-2267.47	-2267.66
AIC	4625.97	4562.95	4561.32
Adjusted R square	0.53	0.595	0.597
Lag (Rho) Coefficient		0.317	
Error (Lambda) Coefficient			0.406
**(b) Overweight**			
Urban	0.004 (0.597)	0.007 (0.227)	0.005 (0.022)
Richest	-0.018 (0.057)	-0.012 (0.162)	-0.011 (0.379)
Scheduled Castes/Tribes	0.035 (0.000)	0.023 (0.000)	0.026 (0.000)
Ten or more years of Schooling	0.002 (0.805)	0.005 (0.589)	0.0097 (0.414)
Exposure to Mass Media	-0.015 (0.141)	-0.014 (0.135)	-0.019 (0.104)
Muslim	0.031 (0.000)	0.019 (0.003)	0.029 (0.001)
Female Child	0.072 (0.011)	0.059 (0.026)	0.049 (0.057)
Three or more Birth Order	-0.049 (0.000)	-0.028 (0.034)	-0.021 (0.218)
Children under 6 months exclusively breastfed	0.003 (0.371)	0.004 (0.322)	0.003 (0.460)
Children 6-23 months receiving an adequate diet	-0.009 (0.572)	-0.009 (0.507)	0.000 (0.999)
Women with Body Mass Index<18.5 kg/m2	-0.149 (0.000)	-0.103 (0.000)	-0.127 (0.000)
Births delivered by Caesarean Section	0.011 (0.304)	0.008 (0.369)	0.022 (0.079)
Constant	3.55 (0.027)	1.58 (0.284)	3.41 (0.022)
Number of Observations	707	707	707
Log likelihood	-1724.98	-1682.25	-1685

AIC	3475.96	3392.5	3396.01
Adjusted R square	0.26	0.378	0.38
Lag (Rho) Coefficient		0.41	
Error (Lambda) Coefficient			0.46
**(c) Anaemia**			
Rural	-0.018 (0.287)	-0.007 (0.674)	-0.013 (0.484)
Poorest	-0.271 (0.000)	0174 (0.000)	-0.109 (0.005)
Scheduled Castes/Tribes	0.034 (00.106)	0.023 (0.162)	0.001 (0.951)
Ten or more years of Schooling	-0.124 (0.000)	-0.060 (0.011)	-0.068 (0.057)
Exposure to Mass Media	-0.060 (0.116)	-0.017 (0.618)	0.021 (0.605)
Muslim	0.035 (0.084)	0.038 (0.036)	0.046 (0.069)
Female Child	0.248 (0.005)	0.282 (0.000)	0.199 (0.011)
Three or more Birth Order	0.000 (0.991)	0.031 (0.400)	0.064 (0.191)
Children under 6 months exclusively breastfed	0.033 (0.004)	0.028 (0.006)	0.023 (0.024)
Children 6-23 months receiving an adequate diet	-0.215 (0.000)	-0.135 (0.002)	-0.059 (0.219)
Women with Body Mass Index<18.5 kg/m2	0.195 (0.000)	0.084 (0.0611)	0.060 (0.291)
Household with Improved Sanitation	0.110 (0.000)	0.083 (0.001)	-0.085 (0.009)
Anaemic Mothers	0.566 (0.000)	0.465 (0.000)	0.583 (0.000)
Constant	43.69 (0.000)	13.51 (0.012)	30.22 (0.000)
Number of Observations	707	707	707
Log likelihood	-2491.62	-2438.06	-2449.29
AIC	5011.25	4906.12	4926.59
Adjusted R square	0.57	0.64	0.64
Lag (Rho) Coefficient		0.36	
Error (Lambda) Coefficient			0.55

Table S2. Result of quantile regression with proportion of children who were stunted, underweight and anaemia by some selected characteristics in 707 districts of India, NFHS-5 (2019–2021). *** $p < 0.001$, ** $p < 0.05$, * $p < 0.1$.

**Childhood Stunting**	**q25**	**q50**	**q75**	**q90**
Rural	-0.0437***	-0.0213	-0.0222	-0.00588
Poorest	-0.000407	-0.00157	0.0377	0.0205
Scheduled Castes/Tribes	0.0154	0.0128	-0.0086	-0.0169
Ten or more years of Schooling	0.029	-0.0109	-0.017	-0.0224
Exposure to Mass Media	-0.0805	-0.0375	-0.0111	0.0117
Muslim	0.0206	0.0274	0.0165	0.0213
Female Child	-0.0206	0.0977	0.0909	0.0947
Three or more Birth Order	0.252***	0.295***	0.372***	0.389***
Children under 6 months exclusively breastfed	0.0153	0.0105	0.0148	-0.0129
Children 6-23 months receiving an adequate diet	-0.0947	-0.0278	-0.0157	-0.0312
Women with Body Mass Index<18.5 kg/m2	0.305***	0.390***	0.342***	0.310***
Household with Improved Sanitation	-0.122***	-0.0708**	-0.0422	-0.0491
Constant	36.28***	24.40**	23.91***	27.30***
**Childhood Overweight**	**q25**	**q50**	**q75**	**q90**
Urban	0.00565	0.00683	0.0137	0.00905
Richest	-0.0132	-0.0194**	-0.0341**	-0.0506*
Scheduled Castes/Tribes	0.00878	0.0261***	0.0513***	0.0514***
Ten or more years of Schooling	-0.00245	0.0196*	0.0299*	0.000993
Exposure to Mass Media	-0.00213	-0.0116	-0.0136	0.000273
Muslim	0.0101	0.0281*	0.0411***	0.0247*
Female Child	0.0173	0.0487	0.0990*	0.155*
Three or more Birth Order	-0.00903	-0.0236	-0.0635*	-0.102**
Children under 6 months exclusively Breastfed	-0.00264	-0.000952	-0.000321	0.00437
Children 6-23 months receiving an adequate diet	0.0238	0.00363	0.0195	0.0038
Women with Body Mass Index<18.5 kg/m2	-0.0870***	-0.123***	-0.192***	-0.266***
Births delivered by Caesarean Section	0.0161	0.0125	-0.00922	-0.0107
Constant	0.328	-0.459	-1.687	0.506
**Childhood Anaemia**	**q25**	**q50**	**q75**	**q90**
Rural	-0.0118	-0.0563	-0.0509*	-0.0225
Poorest	-0.351***	-0.260***	-0.233***	-0.259***
Scheduled Castes/Tribes	0.0184	0.0278	0.0488	0.0702**
Ten or more years of Schooling	-0.195***	-0.146***	-0.181***	-0.174***
Exposure to Mass Media	-0.140*	-0.0896	-0.0756	-0.064
Muslim	0.029	0.0231	0.0144	0.0249
Female Child	-0.0029	0.148	0.0804	-0.0445
Three or more Birth Order	-0.114	-0.0930*	-0.147**	-0.122*

Children under 6 months exclusively Breastfed	0.0451**	0.0392**	0.0286	0.0258
Children 6-23 months receiving an adequate diet	-0.191*	-0.164**	-0.257***	-0.221***
Women with Body Mass Index<18.5 kg/m2	0.217**	0.109	0.0646	0.0638
Household with Improved Sanitation	-0.155***	-0.175***	-0.152***	-0.145***
Anaemic Mothers	0.485***	0.528***	0.472***	0.469***
Constant	71.29***	65.26***	77.62***	82.85***
